# COVID-19 and Other Underlying Causes of Cancer Deaths — United States, January 2018–July 2022

**DOI:** 10.15585/mmwr.mm7150a3

**Published:** 2022-12-16

**Authors:** S. Jane Henley, Nicole F. Dowling, Farida B. Ahmad, Taylor D. Ellington, Manxia Wu, Lisa C. Richardson

**Affiliations:** ^1^Division of Cancer Prevention and Control, National Center for Chronic Disease Prevention and Health Promotion, CDC; ^2^National Center for Health Statistics, CDC; ^3^Oak Ridge Institute for Science and Education, Oak Ridge, Tennessee.

Cancer survivors (persons who have received a diagnosis of cancer, from the time of diagnosis throughout their lifespan)* have increased risk for severe COVID-19 illness and mortality (*1*). This report describes characteristics of deaths reported to CDC’s National Vital Statistics System (NVSS), for which cancer was listed as the underlying or a contributing cause (cancer deaths) during January 1, 2018–July 2, 2022. The underlying causes of death, including cancer and COVID-19, were examined by week, age, sex, race and ethnicity, and cancer type. Among an average of approximately 13,000 weekly cancer deaths, the percentage with cancer as the underlying cause was 90% in 2018 and 2019, 88% in 2020, and 87% in 2021. The percentage of cancer deaths with COVID-19 as the underlying cause differed by time (2.0% overall in 2020 and 2.4% in 2021, ranging from 0.2% to 7.2% by week), with higher percentages during peaks in the COVID-19 pandemic. The percentage of cancer deaths with COVID-19 as the underlying cause also differed by the characteristics examined, with higher percentages observed in 2021 among persons aged ≥65 years (2.4% among persons aged 65–74 years, 2.6% among persons aged 75–84 years, and 2.4% among persons aged ≥85 years); males (2.6%); persons categorized as non-Hispanic American Indian or Alaska Native (AI/AN) (3.4%), Hispanic or Latino (Hispanic) (3.2%), or non-Hispanic Black or African American (Black) (2.5%); and persons with hematologic cancers, including leukemia (7.4%), lymphoma (7.3%), and myeloma (5.8%). This report found differences by age, sex, race and ethnicity, and cancer type in the percentage of cancer deaths with COVID-19 as the underlying cause. These results might guide multicomponent COVID-19 prevention interventions and ongoing, cross-cutting efforts to reduce health disparities and address structural and social determinants of health among cancer survivors, which might help protect those at disproportionate and increased risk for death from COVID-19.

Final mortality data for 2018–2020 and provisional mortality data for 2021–2022, reported to NVSS as of September 4, 2022, were used to assess deaths occurring among U.S. residents in the 50 states and District of Columbia during January 1, 2018–July 2, 2022.^†^ The underlying cause of death and any contributing causes were coded according to the *International Classification of Diseases, Tenth Revision* (ICD-10) (*2*). A single underlying cause of death is listed on the death certificate as the disease or injury initiating the chain of morbid events leading directly to death. Other diseases or conditions might be listed as contributing causes of death if they increased susceptibility to or exacerbated an existing disease or contributed to death in some way but did not initiate the chain of events leading to death.^§^ Cancer deaths were defined as those with malignant neoplasm (ICD-10 codes C00–C97) listed as either the underlying or a contributing cause of death. The weekly numbers of cancer deaths, and their underlying causes, were tabulated.^¶^ The percentages (and 95% Wilson CIs) of cancer deaths with cancer or COVID-19 as the underlying cause of death were examined by year, age, sex, race and ethnicity, and cancer type.** This activity was reviewed by CDC and was conducted consistent with applicable federal law and CDC policy.^††^

On average, approximately 13,000 deaths each week listed cancer as an underlying or contributing cause (range = 12,221–14,845) during January 7, 2018–July 2, 2022, with peaks occurring in January 2021 (14,284) and January 2022 (14,845) (Figure 1) (Supplementary Table, https://stacks.cdc.gov/view/cdc/122581). Approximately 11,500 cancer deaths with cancer as the underlying cause occurred each week during this period, ranging from 10,891 in June 2020 to 12,408 in January 2018. From 2018 to 2021, the annual number of cancer deaths increased 4.7%, and the number with cancer as the underlying cause increased 1.0%. During 2020–2022, the weekly number of cancer deaths with COVID-19 as the underlying cause ranged from 28 to 1,055, peaking in January 2021 (953) and January 2022 (1,055). The weekly number of cancer deaths with COVID-19 as a contributing cause ranged from 10 to 463 during 2020–2022 and was highest in January 2021 (242) and January 2022 (463).

**FIGURE 1:**
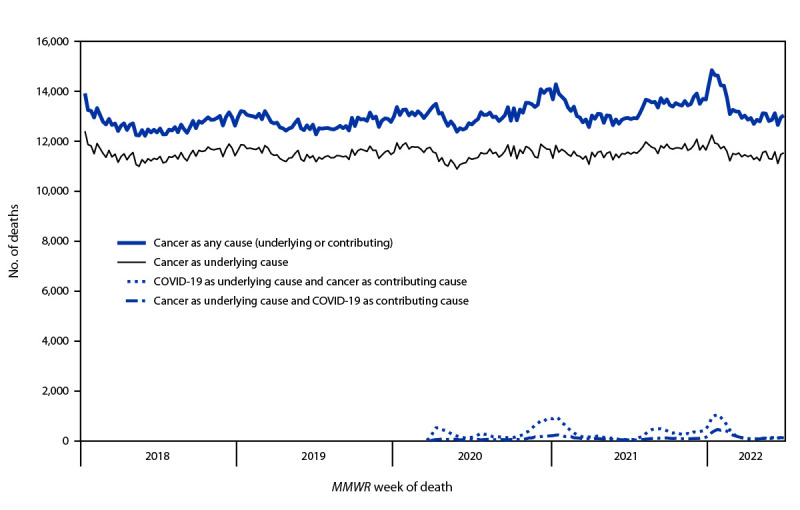
Number* of cancer deaths^†^ with cancer or COVID-19^§^ as underlying or contributing cause of death, by MMWR week of death — United States, January 7, 2018–July 2, 2022 **Abbreviation**: ICD-10 *= International Classification of Diseases, Tenth Revision.* * National Vital Statistics System data for 2018–2020 are final. Provisional data for 2021 and 2022 are incomplete. These data exclude deaths that occurred in the United States among residents of U.S. territories and foreign countries. Based on records received and processed as of September 4, 2022. ^†^ Deaths with malignant neoplasm (cancer), coded to ICD-10 codes C00–C97, as an underlying or contributing cause of death. ^§^ Deaths with confirmed or presumed COVID-19, coded to ICD-10 code U07.1.

Among cancer deaths, the percentage with cancer as the underlying cause was 90% in 2018 and 2019 (weekly range = 89%–91%), 88% (83%–90%) in 2020, and 87% (83%–89%) in 2021 (Table); during the first half of 2022, this percentage ranged from 81% to 89%. Among deaths with cancer as a contributing cause, common noncancer underlying causes included diseases of the circulatory system, including heart disease and stroke; mental and behavioral disorders and diseases of the nervous system, including Alzheimer disease; endocrine, nutritional, metabolic, and digestive system diseases, including diabetes and cirrhosis; diseases of the respiratory system, including chronic obstructive pulmonary disease, influenza, and pneumonia; and COVID-19 (Figure 2). During November 22, 2020–February 6, 2021, and January 9–February 19, 2022, the number of cancer deaths with COVID-19 as underlying cause exceeded the number for any other underlying cause, except cancer. The percentage of cancer deaths with COVID-19 as the underlying cause was 2.0% in 2020 (weekly range = 0.2%–6.4%) and 2.4% in 2021 (range = 0.4%–6.7%) (Table); during the first half of 2022, this percentage ranged from 1.0% to 7.2%.

**TABLE:** Number* of cancer deaths^†^ and percentage of these deaths with cancer^§^ or COVID-19^¶^ as underlying cause of death, by year, sex, age group, race and ethnicity, and cancer type — United States, 2018–2021

Characteristic	No. of deaths				% of deaths (95% CI)					
	Cancer as underlying or contributing cause				Cancer as underlying cause				COVID-19 as underlying cause and cancer as contributing cause	
	2018**	2019	2020	2021	2018^††^	2019	2020	2021	2020^§§^	2021
**Overall**	**662,636**	**664,763**	**685,859**	**693,782**	**90 (90–91)**	**90 (90–90)**	**88 (88–88)**	**87 (87–87)**	**2.0 (1.9–2.0)**	**2.4 (2.3–2.4)**
**Sex**										
Female	310,566	310,857	319,595	323,598	91 (91–91)	91 (91–91)	89 (89–89)	89 (88–89)	1.7 (1.7–1.8)	2.1 (2.1–2.2)
Male	352,070	353,906	366,264	370,184	90 (90–90)	89 (89–89)	87 (87–87)	86 (86–86)	2.2 (2.1–2.2)	2.6 (2.6–2.7)
**Age group, yrs**										
<1	55	59	62	59	93 (83–97)	93 (84–97)	87 (77–93)	88 (77–94)	—^¶¶^	—
1–4	344	306	325	300	95 (92–97)	93 (90–95)	94 (91–96)	94 (91–96)	—	—
5–14	897	817	836	843	94 (92–95)	95 (93–96)	95 (93–96)	94 (93–96)	—	—
15–24	1,455	1,474	1,385	1,430	94 (93–95)	94 (93–95)	94 (93–95)	93 (91–94)	1.4 (1.0–2.2)	1.5 (1.0–2.3)
25–34	3,907	3,812	3,858	3,936	94 (94–95)	94 (93–95)	93 (92–93)	92 (91–93)	1.1 (0.8–1.4)	1.9 (1.5–2.3)
35–44	11,161	11,269	11,476	12,034	95 (95–96)	95 (94–95)	93 (93–94)	93 (93–93)	1.3 (1.1–1.5)	1.7 (1.4–1.9)
45–54	39,187	37,351	36,938	36,289	95 (95–95)	95 (95–95)	94 (93–94)	92 (92–93)	1.2 (1.1–1.3)	2.1 (1.9–2.2)
55–64	121,157	119,048	119,738	118,602	94 (94–94)	94 (94–94)	92 (92–92)	91 (91–91)	1.3 (1.3–1.4)	2.1 (2.0–2.2)
65–74	183,456	186,016	195,426	201,367	92 (92–92)	92 (92–92)	90 (90–90)	89 (89–89)	1.8 (1.8–1.9)	2.4 (2.4–2.5)
75–84	177,829	180,146	188,349	191,954	89 (89–89)	89 (89–89)	86 (86–87)	86 (86–86)	2.2 (2.2–2.3)	2.6 (2.5–2.7)
≥85	123,176	124,452	127,462	126,958	84 (84–84)	83 (83–84)	80 (80–81)	81 (80–81)	2.7 (2.6–2.8)	2.4 (2.3–2.5)
Race and ethnicity***										
AI/AN, NH	3,304	3,323	3,575	3,708	90 (89–91)	90 (89–91)	85 (84–87)	85 (84–86)	3.3 (2.7–4.0)	3.4 (2.9–4.1)
Asian, NH	18,513	19,113	20,320	21,385	93 (93–93)	93 (92–93)	90 (90–90)	90 (90–91)	1.8 (1.6–2.0)	2.0 (1.8–2.2)
Black or African American, NH	76,389	77,312	80,592	79,983	91 (91–91)	91 (91–91)	88 (87–88)	88 (87–88)	2.6 (2.5–2.7)	2.5 (2.4–2.6)
Hispanic or Latino	45,562	46,876	49,708	51,451	92 (92–93)	92 (92–92)	88 (88–89)	89 (88–89)	3.4 (3.2–3.6)	3.2 (3.0–3.4)
NH/OPI, NH	773	809	868	928	93 (91–94)	93 (91–95)	89 (87–91)	91 (88–92)	2.1 (1.3–3.3)	1.5 (0.8–2.6)
White, NH	513,965	513,319	526,665	532,025	90 (90–90)	90 (90–90)	88 (88–88)	87 (87–87)	1.7 (1.7–1.7)	2.3 (2.2–2.3)
Multiracial, NH	2,693	2,761	2,884	3,034	91 (90–92)	91 (90–92)	89 (88–90)	89 (88–90)	1.4 (1.0–1.9)	2.3 (1.8–2.9)
**Cancer type (ICD-10 code)**										
Bladder (C67)	21,443	21,868	22,644	22,933	85 (84–85)	84 (84–85)	81 (81–82)	81 (81–82)	2.3 (2.1–2.5)	2.4 (2.2–2.6)
Breast (C50)	52,571	52,938	55,068	55,660	86 (86–86)	85 (85–86)	82 (82–83)	82 (81–82)	2.6 (2.4–2.7)	2.8 (2.7–3.0)
Cervix uteri (C53)	4,688	4,687	4,922	5,088	93 (92–93)	93 (92–94)	91 (91–92)	90 (89–91)	0.7 (0.5–0.9)	1.4 (1.1–1.8)
Colon, rectum, and anus (C18–C21)	61,234	61,175	62,803	64,003	90 (90–90)	90 (90–91)	88 (88–88)	88 (88–89)	1.7 (1.6–1.8)	1.9 (1.8–2.0)
Corpus uteri and uterus, part unspecified (C54–C55)	12,706	13,035	13,919	14,214	93 (92–93)	93 (93–93)	91 (90–91)	90 (90–91)	1.4 (1.2–1.6)	1.3 (1.1–1.5)
Esophagus (C15)	16,867	17,480	17,432	17,634	94 (94–94)	94 (94–94)	93 (92–93)	92 (92–93)	1.0 (0.9–1.2)	1.2 (1.0–1.3)
Hematologic cancers (C81–C96)	70,368	70,594	75,577	77,437	86 (86–86)	86 (86–86)	81 (81–81)	78 (78–79)	4.5 (4.4–4.7)	7.0 (6.9–7.2)
Hodgkin disease (C81)	1,508	1,446	1,592	1,636	79 (77–81)	78 (75–80)	75 (73–77)	71 (69–73)	3.0 (2.3–4.0)	6.4 (5.2–7.7)
Kidney and renal pelvis (C64–C65)	16,918	16,919	18,007	17,925	89 (89–90)	89 (88–89)	86 (86–87)	85 (84–85)	2.1 (1.9–2.3)	2.5 (2.3–2.7)
Larynx (C32)	4,885	4,949	5,077	5,212	85 (84–86)	85 (84–86)	82 (81–83)	82 (81–83)	1.9 (1.5–2.3)	2.4 (2.0–2.8)
Leukemia (C91–C95)	28,817	28,777	31,177	31,882	86 (86–87)	86 (86–86)	81 (80–81)	78 (78–79)	5.0 (4.7–5.2)	7.4 (7.1–7.7)
Lip, oral cavity, and pharynx (C00–C14)	12,391	12,793	13,447	14,351	89 (88–89)	89 (89–90)	87 (86–88)	86 (86–87)	1.4 (1.2–1.6)	1.4 (1.3–1.7)
Liver and intrahepatic bile ducts (C22)	30,481	30,898	31,660	32,359	93 (93–93)	93 (92–93)	92 (91–92)	91 (91–92)	1.0 (0.9–1.1)	1.0 (0.9–1.1)
Malignant melanoma of skin (C43)	9,621	9,548	9,906	10,085	89 (89–90)	89 (89–90)	87 (87–88)	86 (86–87)	1.5 (1.3–1.7)	1.8 (1.6–2.1)
Meninges, brain, and other CNS (C70–C72)	17,972	18,084	19,073	18,934	97 (97–97)	97 (97–97)	96 (96–96)	96 (96–96)	0.9 (0.8–1.0)	0.9 (0.7–1.0)
Multiple myeloma and immunoproliferative neoplasms (C88 and C90)	15,542	15,842	16,867	17,024	86 (86–87)	86 (85–86)	81 (81–82)	80 (80–81)	4.7 (4.4–5.1)	5.8 (5.5–6.2)
Non-Hodgkin lymphoma (C82–C85)	25,448	25,490	26,964	27,915	86 (86–86)	86 (86–86)	82 (81–82)	78 (78–79)	3.9 (3.7–4.2)	7.3 (7.0–7.6)
Ovary (C56)	14,943	14,620	14,862	14,859	95 (95–95)	95 (95–96)	94 (94–94)	94 (93–94)	0.9 (0.7–1.0)	1.0 (0.8–1.2)
Pancreas (C25)	47,245	48,250	49,690	50,922	97 (97–97)	97 (97–97)	96 (96–96)	96 (96–96)	0.5 (0.5–0.6)	0.6 (0.5–0.7)
Prostate (C61)	43,442	44,395	48,501	48,472	79 (79–80)	78 (78–79)	74 (74–75)	74 (74–75)	3.8 (3.6–4.0)	3.6 (3.4–3.8)
Stomach (C16)	12,016	12,030	12,377	12,135	95 (94–95)	95 (95–96)	93 (93–94)	93 (93–94)	1.1 (0.9–1.3)	1.2 (1.0–1.4)
Trachea, bronchus, and lung (C33–C34)	153,078	150,898	150,053	149,224	94 (94–94)	94 (93–94)	92 (92–92)	91 (91–92)	1.5 (1.5–1.6)	1.8 (1.8–1.9)

**Abbreviations**: AI/AN = American Indian or Alaska Native; CNS = central nervous system; ICD-10 = *International Classification of Diseases, Tenth Revision*; NH = non-Hispanic; NH/OPI = Native Hawaiian or other Pacific Islander.

* National Vital Statistics System data for 2019–2020 are final. Provisional data for 2021 are incomplete. These data exclude deaths that occurred in the United States among residents of U.S. territories and foreign countries. Based on records received and processed as of September 4, 2022.

^†^ Deaths with malignant neoplasm (cancer), coded to ICD-10 codes C00–C97, as an underlying or contributing cause of death.

^§^ Deaths with cancer, coded to ICD-10 codes C00–C97, as an underlying cause of death.

^¶^ Deaths with cancer, coded to ICD-10 codes C00–C97, as a contributing cause of death and confirmed or presumed COVID-19, coded to ICD-10 code U07.1, as an underlying cause of death.

** The overall weekly range of cancer deaths was 12,221–13,923 during 2018; 12,280–13,212 during 2019; 12,381–14,090 during 2020; and 12,569–14,284 during 2021.

^††^ The overall weekly range of percentage of deaths with cancer as underlying cause was 89%–91% during 2018, 89%–91% during 2019, 83%–90% during 2020, and 83%–89% during 2021.

^§§^ The overall weekly range of percentage of deaths with COVID-19 as underlying cause and cancer as contributing cause was 0.2%–6.4% during 2020, and 0.4%–6.7% during 2021.

^¶¶^ Percentages are not reported for cells with <20 deaths.

*** Race and ethnicity were reported separately on the death certificate and combined for this analysis. Hispanic or Latino persons could be of any race. Deaths of persons with Hispanic or Latino ethnicity “Not Stated” were included in overall counts but were not included in specific racial and ethnic group counts. https://wonder.cdc.gov/wonder/help/mcd-provisional.html#Racial%20Differences

**FIGURE 2:**
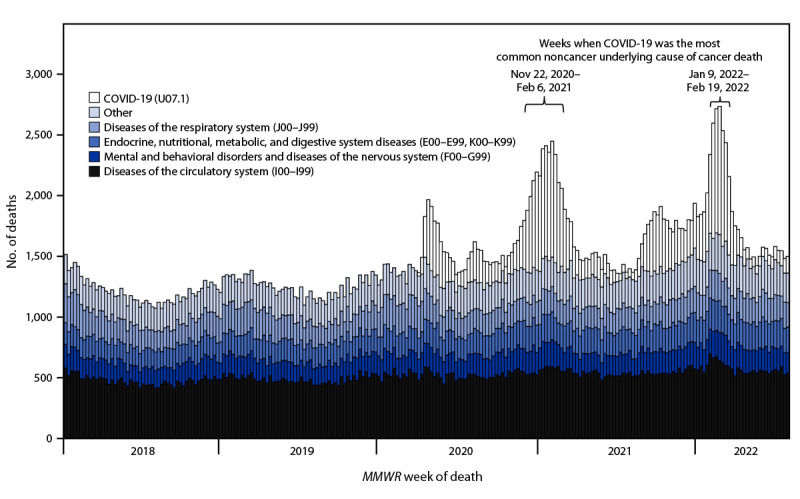
Number* of deaths with cancer as a contributing cause of death,^†^ by noncancer underlying cause of death^§^ and MMWR week of death — United States, January 7, 2018–July 2, 2022 **Abbreviation**: ICD-10 = *International Classification of Diseases, Tenth Revision*. * National Vital Statistics System data for 2018–2020 are final. Provisional data for 2021 and 2022 are incomplete. These data exclude deaths that occurred in the United States among residents of U.S. territories and foreign countries. Based on records received and processed as of September 4, 2022. ^†^ Deaths with malignant neoplasm (cancer), coded to ICD-10 codes C00–C97, as a contributing cause of death. ^§^ Deaths with cancer as a contributing cause of death and the underlying cause of death attributed to other diseases or conditions, including diseases of the circulatory system (ICD-10 codes I00–I99), including heart disease and stroke; mental and behavioral disorders and diseases of the nervous system (F00–G99), including Alzheimer disease; endocrine, nutritional, metabolic, and digestive system diseases (E00–E99, K00–K99), including diabetes and cirrhosis; diseases of the respiratory system (J00–J99), including chronic obstructive pulmonary disease, influenza, and pneumonia; confirmed or presumed COVID-19 (U07.1); and all other causes. Together, these deaths accounted for <20% of all cancer deaths (weekly range = 9%–19%).

The percentage of cancer deaths with COVID-19 as the underlying cause differed by demographic characteristics and type of malignancy. In 2021, a higher percentage of cancer deaths with COVID-19 as the underlying cause occurred among males (2.6%) than females (2.1%); persons aged ≥65 years (2.4% among persons aged 65–74 years, 2.6% among persons aged 75–84 years, and 2.4% among persons aged ≥85 years) than among those aged 15–64 years (ranging from 1.5% to 2.1% by age group); and AI/AN persons (3.4%), Hispanic persons (3.2%), and Black persons (2.5%) compared with a range from 1.5% to 2.3% among persons of other racial and ethnic groups. A higher percentage of hematologic cancer deaths had COVID-19 as the underlying cause (7.4% of leukemia, 7.3% of non-Hodgkin lymphoma, and 5.8% of myeloma deaths) compared with 0.6% of pancreatic cancer deaths, 2.8% of breast cancer deaths, and 3.6% of prostate cancer deaths.

## Discussion

Cancer was one of the first conditions to be linked with increased risk for severe COVID-19 morbidity and mortality (*1*). This report showed that the number of cancer deaths with cancer as the underlying cause increased slightly from 2018 to 2021, but relatively less than the increase in the number of deaths from cancer as any cause of death, indicating that an excess number of persons with cancer died from COVID-19 and other diseases. The number of cancer deaths that were due to noncancer underlying conditions was highest during winter months in 2021 and 2022, which correspond to peaks in COVID-19 infection.^§§^ Whereas many of these cancer deaths listed COVID-19 as the underlying cause, other cancer deaths during this time might have had underlying conditions (e.g., heart disease) exacerbated by unreported COVID-19 illness or underlying conditions (e.g., drug overdose or cirrhosis) exacerbated by changes in health behaviors during the pandemic (*3*).

Some persons might be moderately or severely immunocompromised because of their cancer or cancer treatment, such as active treatment for solid tumors or blood cancers or high-dose corticosteroids or other drugs that suppress the immune system.^¶¶^ Because hematologic cancers develop in the immune system, persons living with these cancers tend to have weakened immune systems and might be particularly susceptible to COVID-19 infection and disease progression (*4*). This report found that a disproportionately high percentage of persons with leukemia, lymphoma, myeloma, and other hematologic cancers died from COVID-19.

Up-to-date COVID-19 vaccination reduces the risk of severe COVID-19 illness (*5*). Additional doses in the primary series and boosters are generally recommended for persons who are moderately or severely immunocompromised.*** Health care providers can inform their cancer patients about the recommended COVID-19 vaccination series and the timing of COVID-19 vaccination administration relative to their cancer treatment (*6*). Up-to-date COVID-19 vaccination for close contacts has been shown to protect cancer patients from infection (*7*). Other interventions, such as mask use, physical distancing, good hand hygiene, and adequate indoor ventilation, are shown to prevent infection.^†††^ Some cancer patients might benefit from monoclonal antibodies as preexposure prophylaxis or from anti–SARS-CoV-2 therapies such as Paxlovid and molnupiravir (*7*).

This report found a disproportionately high percentage of cancer deaths with COVID-19 as the underlying cause among Hispanic, AI/AN, and Black persons compared with the percentage in other racial and ethnic groups. Similar disparities have been observed for COVID-19 mortality (*8*) as well as cancer mortality (*9*). Health inequities are driven, in part, by structural racism, discrimination, stigma, and longstanding disenfranchisement (*10*). CDC is collaborating with local, state, tribal, and national partners to address environmental, place-based, occupational, policy, and systemic factors that affect health outcomes.^§§§^ For example, national cancer programs funded by CDC are required to include activities to identify drivers of cancer health disparities and address inequities in populations disproportionately affected by the increased risk for cancer or by the lack of adequate health care options for prevention or treatment.^¶¶¶^ Disproportionately affected populations can be defined by sex, race, religion, ethnicity, culture, disability, sexual orientation, gender identity, geographic location, socioeconomic status, insurance status, literacy level, or the intersection of several of these factors that collectively affect health outcomes.

The findings in this report are subject to at least three limitations. First, 2021 and 2022 data are provisional, and numbers might change as additional information is received. Second, ethnicity, race, or both might have been inaccurately recorded on death certificates,**** which might result in under- or overestimates of death counts in some groups. Finally, information about cancer diagnosis that might be related to prognosis, such as date of diagnosis, screening status, treatment status, or barriers to cancer care, was not available in the death certificate; some cancer survivors might have been in treatment when they died, whereas others might have had a remote history of cancer.

This report found disproportionately higher percentage of cancer deaths with COVID-19 as the underlying cause of death among persons who were older; male; categorized as Hispanic, AI/AN, and Black; or living with certain cancers, such as leukemia, lymphoma, and myeloma. These results could guide multicomponent COVID-19 prevention interventions and ongoing, cross-cutting efforts to reduce health disparities and address structural and social determinants of health among cancer survivors, which might help protect those at disproportionately increased risk for dying from COVID-19.

**Summary**

**What is already known about this topic?**

Persons with cancer are at increased risk for dying from COVID-19.

**What is added by this report?**

Among persons who died with cancer, 2.0% in 2020 and 2.4% in 2021 had COVID-19 listed as the underlying cause of death, with higher percentages during COVID-19 peaks and among persons who were older, male, Hispanic or Latino, non-Hispanic American Indian or Alaska Native, non-Hispanic Black or African American, or living with leukemia, lymphoma, or myeloma.

**What are the implications for public health practice?**

These results might guide COVID-19 prevention interventions and efforts focusing on reducing health disparities and addressing structural and social determinants of health among cancer survivors, which might help protect those at disproportionately increased risk for dying from COVID-19.

## Footnotes

*
https://www.cdc.gov/cancer/ncccp/priorities/cancer-survivor-caregiver.htm

† NVSS final and provisional mortality data are available at https://wonder.cdc.gov. Data were obtained from the CDC WONDER Provisional Multiple Cause of Death data file based on records received and processed as of September 4, 2022.

§ https://www.cdc.gov/nchs/nvss/revisions-of-the-us-standard-certificates-and-reports.htm; https://www.cdc.gov/nchs/data/nvss/vsrg/vsrg03-508.pdf; https://www.cdc.gov/nchs/pressroom/podcasts/2022/20220107/20220107.htm

¶ Underlying cause of death was coded as follows by using ICD-10: malignant neoplasms (cancer) (ICD-10 codes C00–C97); diseases of the circulatory system (I00–I99), including heart disease and stroke; mental and behavioral disorders and diseases of the nervous system (F00–G99), including Alzheimer disease; endocrine, nutritional, metabolic, and digestive system diseases (E00–E99 and K00–K99), including diabetes and cirrhosis; diseases of the respiratory system (J00–J99), including chronic obstructive pulmonary disease, influenza, and pneumonia; confirmed or presumed COVID-19 (U07.1); and all other causes.

** Race and ethnicity were reported separately on the death certificate and combined for this analysis. https://wonder.cdc.gov/wonder/help/mcd-provisional.html#Racial%20Differences

†† 45 C.F.R. part 46, 21 C.F.R. part 56; 42 U.S.C. Sect. 241(d); 5 U.S.C. Sect. 552a; 44 U.S.C. Sect. 3501 et seq.

§§
https://covid.cdc.gov/covid-data-tracker/?CDC_AA_refVal = https%3A%2F%2Fwww.cdc.gov%2Fcoronavirus%2F2019-ncov%2Fcases-updates%2Fcases-in-us.html#trends_dailycases

¶¶
https://www.cdc.gov/coronavirus/2019-ncov/need-extra-precautions/people-with-medical-conditions.html

***
https://www.cdc.gov/coronavirus/2019-ncov/vaccines/recommendations/immuno.html

†††
https://www.cdc.gov/coronavirus/2019-ncov/prevent-getting-sick/prevention.html

§§§ https://www.cdc.gov/healthequity/core/index.html; https://www.cdc.gov/coronavirus/2019-ncov/community/health-equity/index.html

¶¶¶ https://www.cdc.gov/media/releases/2022/p0608-cancer-award.html; https://www.cdc.gov/cancer/health-equity/

****
https://www.cdc.gov/nchs/data/series/sr_02/sr02_172.pdf
